# Neurotrophin and Wnt signaling cooperatively regulate dendritic spine formation^☆^

**DOI:** 10.1016/j.mcn.2013.04.006

**Published:** 2013-09

**Authors:** Brian G. Hiester, Domenico F. Galati, Patricia C. Salinas, Kevin R. Jones

**Affiliations:** a Department of Molecular, Cellular and Developmental Biology, 347 UCB, University of Colorado, Boulder, CO 80309, United States; b Department of Cell and Developmental Biology, University College London, London WC1E 6BT, United Kingdom

**Keywords:** Wnt signaling, BDNF, Dendritic spine, Dendrite branching, Synapse

## Abstract

Dendritic spines are major sites of excitatory synaptic transmission and changes in their numbers and morphology have been associated with neurodevelopmental and neurodegenerative disorders. Brain-derived Neurotrophic Factor (BDNF) is a secreted growth factor that influences hippocampal, striatal and neocortical pyramidal neuron dendritic spine density. However, the mechanisms by which BDNF regulates dendritic spines and how BDNF interacts with other regulators of spines remain unclear. We propose that one mechanism by which BDNF promotes dendritic spine formation is through an interaction with Wnt signaling. Here, we show that Wnt signaling inhibition in cultured cortical neurons disrupts dendritic spine development, reduces dendritic arbor size and complexity, and blocks BDNF-induced dendritic spine formation and maturation. Additionally, we show that BDNF regulates expression of Wnt2, and that Wnt2 is sufficient to promote cortical dendrite growth and dendritic spine formation. Together, these data suggest that BDNF and Wnt signaling cooperatively regulate dendritic spine formation.

## Introduction

Dendritic spines, specialized protrusions emanating from neuronal dendritic shafts, are the postsynaptic sites of most excitatory synapses in the central nervous system (Harris and Kater, 1994). Dendritic spines first develop as dynamic filopodia that can then undergo expansion of the spine head in a process central to the establishment of excitatory neural synapses (Arellano et al., 2007; Fiala et al., 1998; Ziv and Smith, 1996). Formation, loss, and structural plasticity of dendritic spines have been proposed to underlie experience-dependent changes in brain circuitry during development and in the adult (Alvarez and Sabatini, 2007; Yuste and Bonhoeffer, 2001). Related, perturbations in the regulation of dendritic spines may contribute to the pathophysiology of neurodevelopmental and neurodegenerative disorders, many of which are accompanied by dendritic spine abnormalities (Chapleau et al., 2009; Penzes et al., 2011). Although many different molecular signals have been identified that regulate the formation of dendritic spines (Lin and Koleske, 2010), our understanding of the mechanisms underlying dendritic spine formation is incomplete.

The neurotrophin family of secreted signaling molecules regulates synapse and dendritic spine formation (Huang and Reichardt, 2001; Waterhouse and Xu, 2009). In particular, Brain-derived Neurotrophic Factor (BDNF) signaling is required for dendritic spine formation in several brain regions including the cortex (Chakravarthy et al., 2006; English et al., 2012; Kaneko et al., 2012; Vigers et al., 2012), hippocampus (Luikart et al., 2005; Tyler and Pozzo-Miller, 2003; von Bohlen und Halbach et al., 2008) and striatum (Baquet et al., 2004; Rauskolb et al., 2010). BDNF specifically promotes the maturation of dendritic spines, and has been shown to increase dendritic spine head width in hippocampal neurons (Chapleau et al., 2009; Tyler and Pozzo-Miller, 2003; Verpelli et al., 2010). Interestingly, BDNF signaling is required for activity-induced dendritic spine head enlargement (Tanaka et al., 2008).

Recent research has begun to elucidate some of the molecular mechanisms by which BDNF regulates dendritic spine formation and stability. BDNF-induced dendritic spine formation is dependent on BDNF-mediated regulation of surface expression of TRPC3 voltage-gated calcium channels (Amaral and Pozzo-Miller, 2007) and BDNF-mediated activation of ERK1/2 signaling cascades (Alonso et al., 2004). BDNF-induced spine remodeling also requires Ryanodine receptor-mediated calcium release (Adasme et al., 2011). Additionally, BDNF regulates the trafficking and synaptic localization of PSD95 (Yoshii and Constantine-Paton, 2007), which is a major postsynaptic scaffolding protein that is sufficient to promote dendritic spine formation (El-Husseini et al., 2000). Furthermore, BDNF increases dendritic spine head width through mechanism that requires the Vav family of guanine nucleotide exchange factors (Hale et al., 2011). However, it is likely that additional downstream effectors mediate BDNF-induced dendritic spine formation and stability.

One potential mechanism by which BDNF may regulate dendritic spine formation is through the regulation of other secreted signaling molecules, initiating a dialog between neurons that leads to synapse formation. In addition to the neurotrophins, other secreted growth factors, including TGF-β, FGFs and Wnts, are known to regulate synapse formation (Packard et al., 2003; Salinas, 2005). How these different secreted signals interact and are coordinated is unclear. To identify targets of BDNF regulation, we performed a microarray analysis of forebrain-specific BDNF conditional knockout mouse striatum (Strand et al., 2007), where dendritic spine formation is dependent on BDNF (Baquet et al., 2004), and found that the expression of several Wnt genes is dysregulated. Interestingly, Nerve Growth Factor (NGF), a neurotrophin related to BDNF, stimulates Wnt5a expression, which in turn stimulates axon branching in developing sympathetic neurons (Bodmer et al., 2009). Additionally, BDNF upregulates Wnt signaling in neural stem cells (Chen et al., 2013), and Wnt signaling has been reported to regulate BDNF expression in retinal glial cells (Yi et al., 2012). Together, these observations indicate that there are multiple regulatory connections between neurotrophin and Wnt signaling pathways.

The Wnts are a family of secreted signaling proteins that regulate numerous aspects of nervous system development (Ciani and Salinas, 2005) including neural patterning, neural precursor proliferation and differentiation, and cell migration (Freese et al., 2010). Importantly, studies have shown that Wnts function as key synaptic organizing factors in the vertebrate and invertebrate nervous systems (Budnik and Salinas, 2011; Lu and Van Vactor, 2007). In the mammalian CNS, Wnts promote the presynaptic assembly of cerebellar synapses by signaling in a retrograde manner (Ahmad-Annuar et al., 2006). In addition, Wnt signaling enhances hippocampal excitatory neurotransmission (Cerpa et al., 2008, 2011; Varela-Nallar et al., 2010) by regulating presynaptic (Davis et al., 2008; Farias et al., 2007; Varela-Nallar et al., 2009) and postsynaptic (Ciani et al., 2011; Farias et al., 2009) assembly, and inhibiting Wnt signaling induces hippocampal excitatory synapse disassembly (Davis et al., 2008; Purro et al., 2012). Furthermore, Wnt signaling is a critical component of activity-mediated synapse formation in the hippocampus (Chen et al., 2006; Gogolla et al., 2009; Sahores et al., 2010). Most recently, Wnt signaling has been shown to promote hippocampal dendritic spine formation. Two individual Wnts, Wnt5a and Wnt7a, increase dendritic spine density in cultured hippocampal neurons (Ciani et al., 2011; Farias et al., 2009). Additionally, cultured hippocampal neurons from *Dvl1* knockout mice form fewer dendritic spines (Ciani et al., 2011). Despite the growing appreciation for the role of Wnt signaling in regulating synapse and dendritic spine formation in the cerebellum and hippocampus, a function for Wnt signaling during cortical synapse or dendritic spine formation has not been described.

We hypothesized that one mechanism by which BDNF regulates cortical dendritic spine formation is by specifically regulating members of the Wnt family of secreted signaling proteins. Here, we describe evidence suggesting that Wnt signaling is needed for a subset of BDNF-induced effects on cortical dendrites, particularly the formation and maturation of dendritic spines. Additionally, we present evidence indicating that one specific target of BDNF regulation is Wnt2, and we show that Wnt2 can induce dendritic spine formation in cortical neurons.

## Results

### Wnt inhibition impairs cortical dendritic spine formation and decreases dendrite growth

To investigate whether Wnt signaling is required for dendrite development in cortical neurons, we used four different Wnt inhibitors, Wif1, Sfrp1, mFzd8CRD-IgG and mDvl1ΔPDZ. Wnts signal through both canonical and non-canonical signaling cascades (Logan and Nusse, 2004), and these inhibitors can interfere with both types of Wnt signaling cascades. Wnt-inhibitory Factor-1 (Wif1) and Secreted Frizzled-Related Protein-1 (Sfrp1) are endogenous secreted proteins that can bind to Wnt ligands in the extracellular space and prevent them from binding their receptor (Malinauskas et al., 2011; Rattner et al., 1997). mFzd8CRD-IgG is a secreted fusion protein consisting of the extracellular domain of the murine Wnt receptor Frizzled-8 fused to the human immunoglobulin light chain. mFzd8CRD-IgG also binds to Wnt ligands in the extracellular space and prevents them from binding their receptor (Hsieh et al., 1999). mDvl1ΔPDZ is a deletion mutant of the murine Dishevelled-1 protein, an essential intracellular component of both canonical and non-canonical Wnt signaling cascades (Gao and Chen, 2010). mDvl1ΔPDZ lacks the PDZ domain that is required for Dvl1 to promote hippocampal dendrite growth (Rosso et al., 2005).

**Fig. 1:**
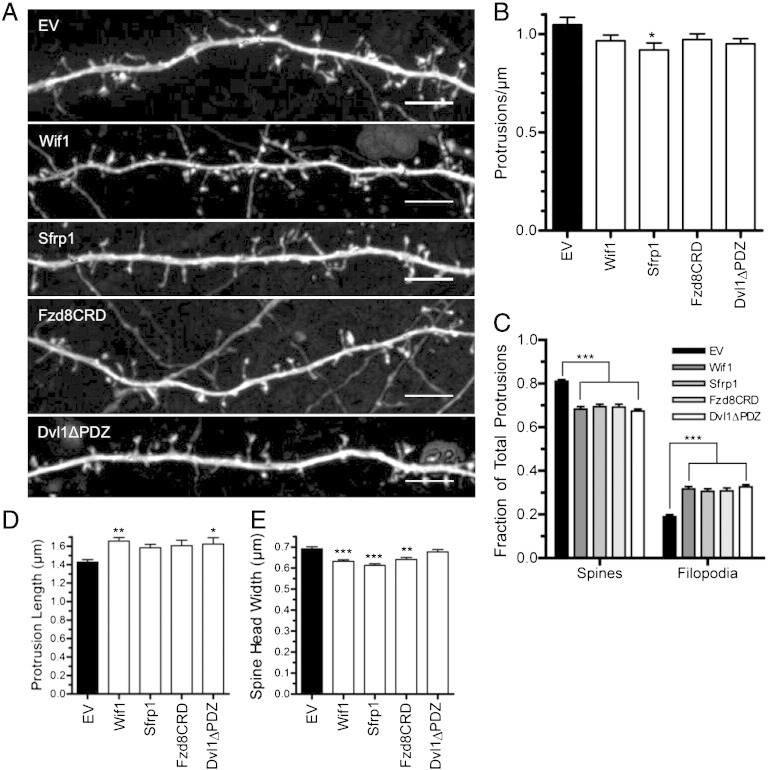
Wnt inhibition results in altered dendritic spine development. (A) Representative dendritic segments of cortical neurons expressing empty vector (EV), Wif1, Sfrp1, Fzd8CRD or Dvl1ΔPDZ. (B) Quantification of dendritic protrusion density. (C) Percent of all dendritic protrusions classified as either spines or filopodia. Quantification of average protrusion length (D) and average spine head width (E) for each treatment. ***p < 0.001, **p < 0.01, *p < 0.05. n = number of neurons: EV n = 31, Wif1 n = 34 Sfrp1 n = 23, Fzd8CRD n = 25, Dvl1ΔPDZ n = 25. Scale bar: 5 μm.

Cultured cortical neurons were co-transfected on DIV10 with a plasmid expressing one of the four different Wnt inhibitors and with a plasmid expressing cytoplasmic GFP in order to visualize neuron morphology. Neurons were then fixed and imaged on DIV14. Representative segments from empty vector (EV) control and Wnt-inhibited neurons are shown in Fig. 1A. Quantitation of total dendritic protrusion density revealed that only Sfrp1 caused a significant decrease (Fig. 1B).

Although total protrusion density was relatively unaffected by Wnt inhibition, morphological classification of dendritic protrusions revealed that all four Wnt inhibitors decreased the percentage of dendritic protrusions classified as spines and increased the percentage of filopodial protrusions (Fig. 1C), suggesting that Wnt inhibition blocks dendritic spine maturation. Dendritic spines are highly plastic structures that undergo dramatic changes in morphology after they emerge and as they mature. New dendritic spines emerge as long filopodia, which, throughout the course of spine maturation increase in spine head width and retract toward the dendritic shaft as the nascent synapse develops (Fiala et al., 1998; Knott et al., 2006; Lohmann and Bonhoeffer, 2008; Maletic-Savatic et al., 1999; Zito et al., 2009). Importantly, increased dendritic spine head volume is associated with increased synaptic strength (Matsuzaki et al., 2004; Yang et al., 2008; Zito et al., 2009), while decreased spine head volume correlates with decreased synaptic strength (Zhou et al., 2004). To further determine whether dendritic spine development was impaired by Wnt inhibition, we measured the length of all dendritic protrusions and the spine head width of dendritic spines. Both Wif1 and Dvl1ΔPDZ caused a significant increase in average protrusion length (Fig. 1D), and relative frequency distribution plots of spine lengths indicate that all four Wnt inhibitors caused a significant decrease in the fraction of short protrusions and a significant increase in the fraction of long protrusions when compared to control (Fig. S4A–D). Importantly, Wif1, Sfrp1 and Fzd8CRD caused significant decreases in dendritic spine head width (Fig. 1E). Together, these data suggest that inhibiting Wnt signaling in cortical neurons impairs dendritic spine formation by inhibiting their maturation.

**Fig. 2:**
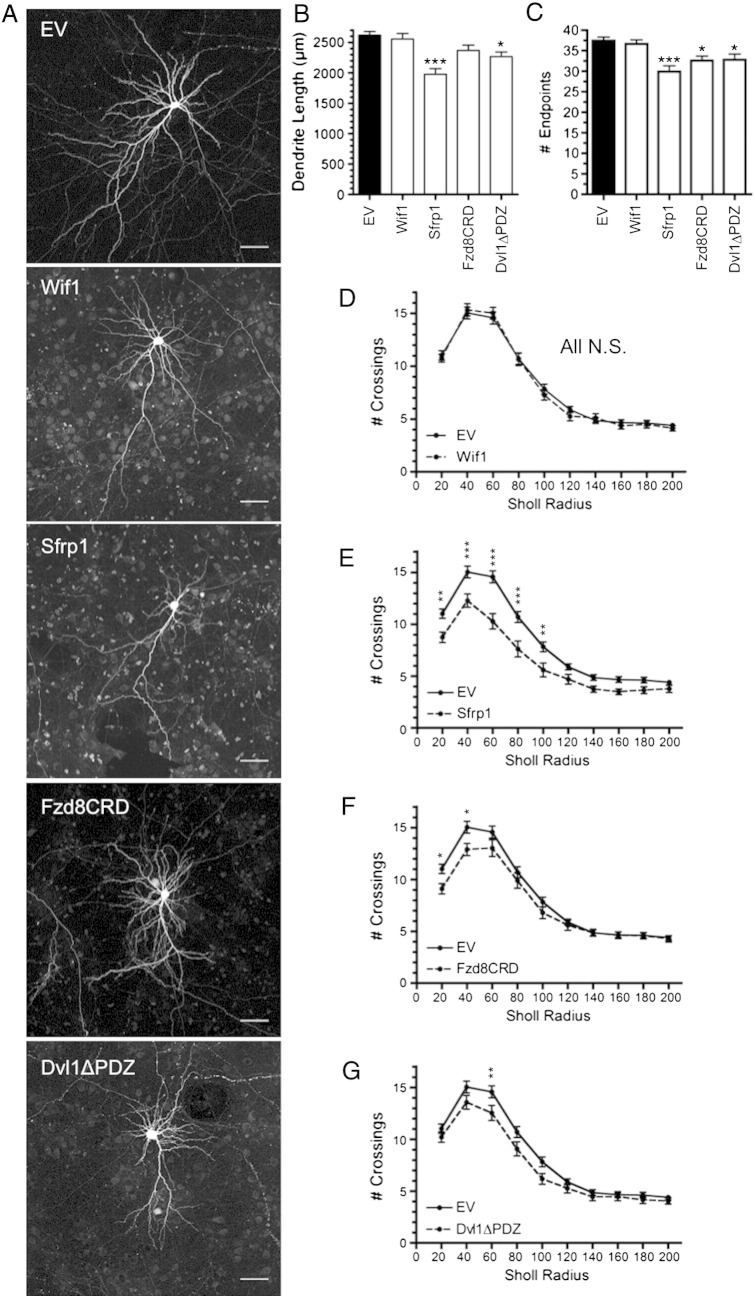
Wnt inhibition results in decreased dendrite elaboration. (A) Representative cortical neurons expressing EV, Wif1, Sfrp1, Fzd8CRD or Dvl1ΔPDZ. (B) Quantification of the total dendrite length per neuron for each treatment. (C) Quantification of the number of dendritic endpoints per neuron for each treatment. (D–G) Sholl analysis of dendritic complexity comparing neurons treated with each Wnt inhibitor to control neurons. ***p < 0.001, **p < 0.01, *p < 0.05. n = number of neurons: EV n = 56, Wif1 n = 49, Sfrp1 n = 39, Fzd8CRD n = 39, Dvl1ΔPDZ n = 39. Scale bar: 50 μm.

Substantial evidence suggests that synaptic activity stabilizes newly formed dendritic branches (Cline and Haas, 2008; Lohmann et al., 2002; Rajan et al., 1999; Vaughn et al., 1988). Both Sfrp1 and Dvl1ΔPDZ inhibit dendritic growth in hippocampal neurons (Rosso et al., 2005), and Wif1 specifically prevents activity-induced hippocampal dendrite growth (Wayman et al., 2006). In order to determine if the defects that we observed in dendritic spine formation due to Wnt inhibition were associated with decreased cortical dendrite arbor size, we measured several aspects of dendrite elaboration: dendrite length, number of dendrite endpoints and total dendritic complexity as determined using a Sholl analysis (Sholl, 1954). Wif1 expression was not sufficient to inhibit any measured aspect of dendrite arborization. However, both Sfrp1 and Dvl1ΔPDZ caused a significant decrease in total dendrite length (Fig. 2B). Additionally, expression of Sfrp1, Fzd8CRD and Dvl1ΔPDZ decreased the number of dendrite endpoints per neuron, suggesting a reduction in total dendritic branching (Fig. 2C). Indeed, Sholl analysis revealed a significant decrease in dendritic complexity for these three treatments (Fig. 2D–F). None of the four Wnt inhibitors caused a decrease in the number of primary dendrites (Fig. S2). Together, these data indicate that some Wnt signaling inhibitors can reduce dendritic arborization in cortical neurons.

### Wnt signaling inhibitors interfere with BDNF-induced cortical dendritic spine formation

**Fig. S1:**
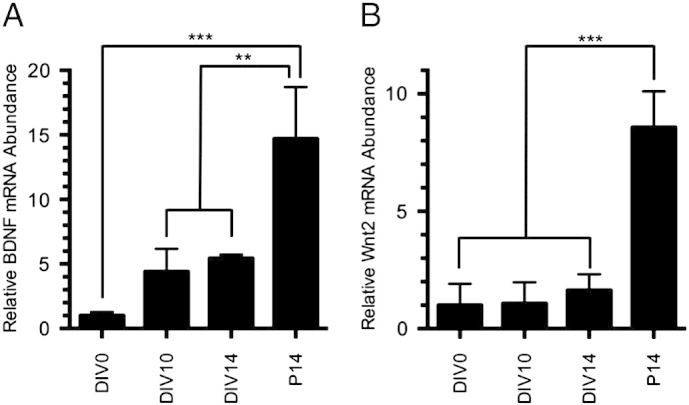
BDNF and Wnt2 mRNA abundances rise during culture of cortical neurons. Quantification of (A) BDNF and (B) Wnt2 mRNA abundances in cultured cortical neurons at DIV0, DIV10 and DIV14, and in mouse neocortex at P14, normalized to 18S RNA. All sample means were divided by the mean at DIV0 in order to determine the relative mRNA expression levels. **p < 0.01, ***p < 0.001. n = number of cell culture wells or number of mice: DIV0 n = 3, DIV10 n = 3, DIV14 n = 3, P14 n = 3.

**Fig. 3:**
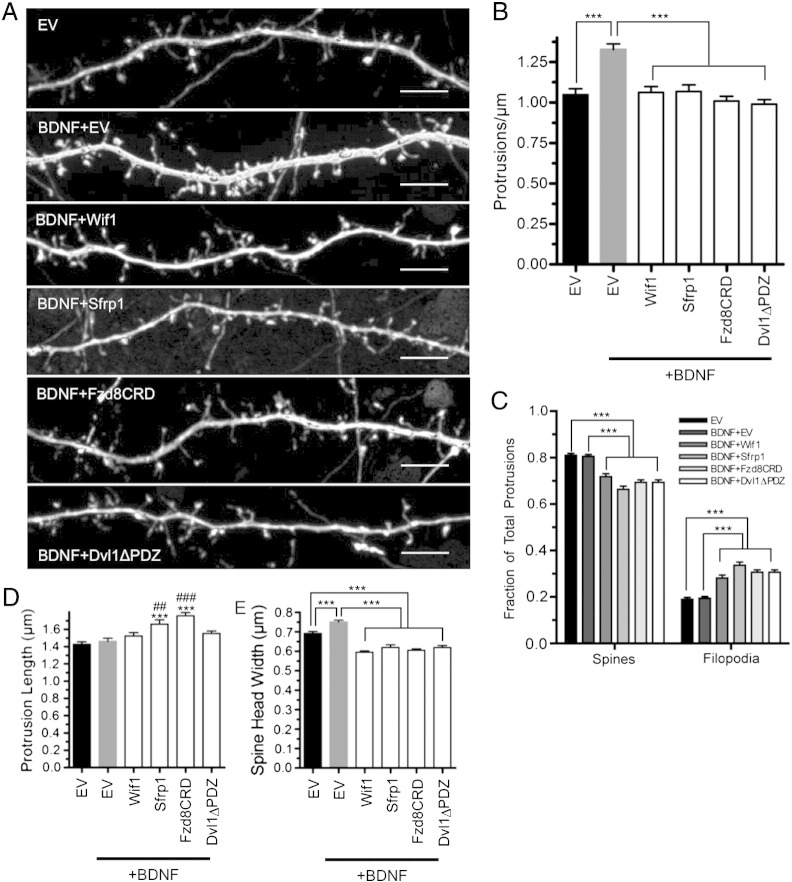
Wnt inhibitors interfere with BDNF-induced dendritic spine formation. (A) Representative dendritic segments of cortical neurons expressing EV, BDNF with EV, BDNF with Wif1, BDNF with Sfrp1, BDNF with Fzd8CRD and BDNF with Dvl1ΔPDZ. (B) Quantification of dendritic protrusion density. (C) Fraction of all spines classified as either spines or filopodia. Quantification of average protrusion length (D) and average spine head width (E) for each treatment. ***p < 0.001, **p < 0.01, *p < 0.5. (* = relative to EV, # = relative to EV + BDNF.) n = number of neurons: EV n = 31, BDNF + EV n = 30, BDNF + Wif1 n = 29, BDNF + Sfrp1 n = 25, BDNF + Fzd8CRD n = 25, BDNF + Dvl1ΔPDZ n = 30. Scale bar: 5 μm.

We next sought to determine the effects of the Wnt signaling inhibitors on BDNF-induced dendritic spine formation. A low level of BDNF expression is present in our cultures, rising during the culture period, reminiscent of the normal *in vivo* postnatal rise of BDNF expression in the cortex, although levels *in vitro* at DIV14 appear lower than present *in vivo* at P14 (Fig. S1A). Notably, expression rises markedly after P14 *in vivo* (Bracken and Turrigiano, 2009; Schoups et al., 1995). Thus, BDNF levels in the cultures even at DIV14 appear substantially lower than those present in the adult cortex, where we have demonstrated that loss of BDNF leads to a loss of dendritic spines (English et al., 2012; Vigers et al., 2012). Against this low background of BDNF, overexpression by transfection of a BDNF-expressing plasmid led to a significant increase in dendritic protrusion density (Fig. 3A). Importantly, each of the four Wnt inhibitors blocked the increase in protrusion density caused by BDNF overexpression (Fig. 3A). Quantification showed that BDNF overexpression increased dendritic protrusion density 27 ± 3%, whereas co-expression of any of the four Wnt inhibitors blocked this increase (Fig. 3B). These data suggest that Wnt signaling is necessary for BDNF-induced dendritic protrusion formation or stabilization.

We next assessed the morphology of protrusions remaining on neurons overexpressing BDNF with each Wnt inhibitor in order to assess how BDNF and Wnt signaling interactions may affect dendritic spine maturation. BDNF overexpression did not affect the fraction of filopodial dendritic protrusions (Fig. 3C) or protrusion length (Fig. 3D). However, BDNF overexpression significantly increased dendritic spine head width, suggesting an enhancement of spine maturation (Fig. 3E). Importantly, this increase was blocked by each of the four Wnt inhibitors (Fig. 3E). Thus, as described above for protrusion density, Wnt inhibitors blocked a significant effect of BDNF on spine maturation.

BDNF overexpression appeared to further interact with the Wnt inhibitors in shaping spine morphology. BDNF co-expression appeared to accentuate the increased protrusion length caused by Sfrp1 and Fzd8CRD (compare Figs. 3D and 1D). Further, BDNF co-expression appeared to accentuate the decrease in spine head width caused by Dvl1ΔPDZ (compare Figs. 3E and 1E). Lastly, BDNF co-expression with each of the Wnt inhibitors was not sufficient to rescue the increased percentage of filopodial spines due to Wnt inhibition (Fig. 3B). Collectively, these results suggest that Wnt inhibition impairs dendritic spine maturation both in the presence and absence of increased BDNF expression.

**Fig. S2:**
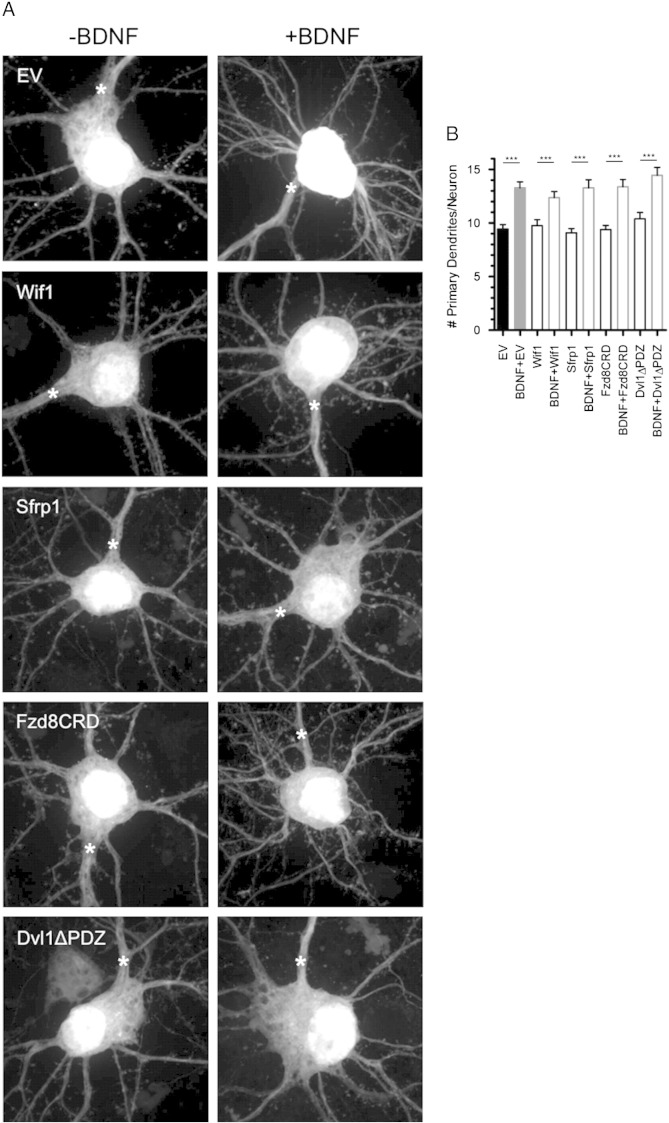
Wnt inhibition does not affect primary dendrite number on cortical neurons. (A) Representative cell bodies of cortical neurons expressing EV, Wif1, Sfrp1, Fzd8CRD and Dvl1ΔPDZ either alone, or in combination with BDNF. (B) Quantification of the number of primary dendrites per neuron for each treatment. ***p < 0.001. n = number of neurons: EV n = 31, BDNF n = 31, Wif1 n = 34, BDNF + Wif1 n = 29, Sfrp1 n = 22, BDNF + Sfrp1 n = 21, Fzd8CRD n = 25, BDNF + Fzd8CRD n = 25, Dvl1ΔPDZ n = 25, BDNF + Dvl1ΔPDZ n = 30. Scale bar: 5 μm.

**Fig. S3:**
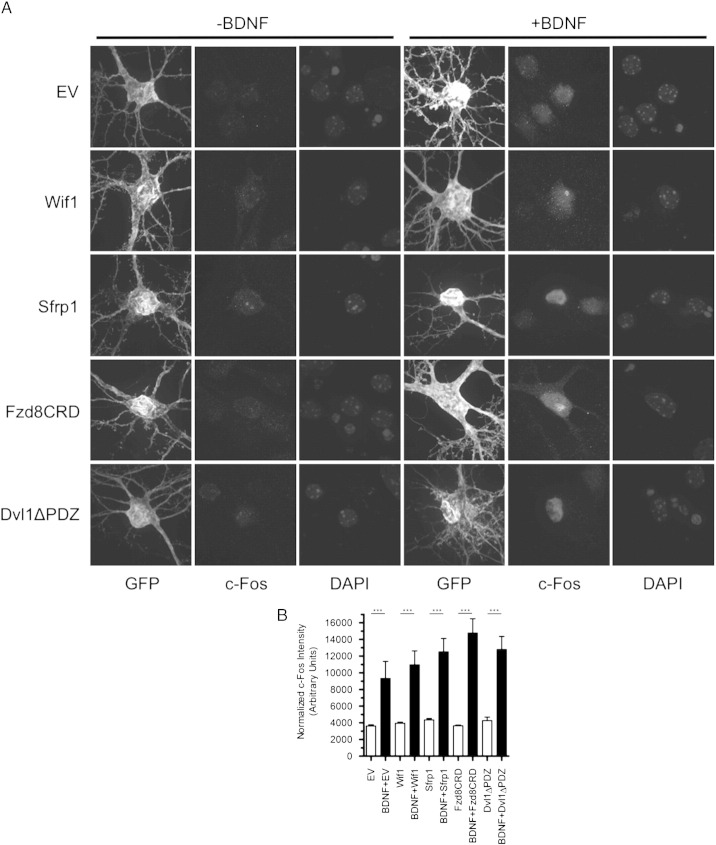
Transfection with Wnt inhibitors does not prevent BDNF-induced c-Fos expression in cortical neurons. (A) GFP fluorescence, c-Fos immunoreactivity and DAPI fluorescence of representative cell bodies of cortical neurons expressing EV, Wif1, Sfrp1, Fzd8CRD and Dvl1ΔPDZ either alone, or in combination with BDNF. (B) Quantification of normalized nuclear c-Fos immunoreactivity per neuron for each treatment. ***p < 0.001. n = 10 neurons for each treatment.

BDNF overexpression rapidly and robustly increases primary dendrite formation in cortical neurons (Horch et al., 1999; McAllister et al., 1997; Wirth et al., 2003). We reproduced this finding, and found that this increase was not blocked by overexpression of the Wnt inhibitors (Fig. S2), indicating that some aspects of BDNF modulation of dendrites remain intact in the presence of Wnt inhibitors. To further assess whether expression of the Wnt inhibitors impaired the signaling ability of BDNF, we analyzed autocrine induction of c-Fos expression by BDNF overexpression. c-Fos is an immediate early gene whose transcription is rapidly upregulated by BDNF (Calella et al., 2007; Gaiddon et al., 1996). We found that BDNF induced c-Fos expression was not reduced in neurons overexpressing any of the four Wnt inhibitors, suggesting that the ability of the inhibitors to interfere with BDNF-induced spine formation and spine head width expansion was not a result of decreased levels of BDNF signaling (Fig. S3).

### BDNF regulation of Wnt2 expression

Our microarray analysis indicated that Wnt2 expression is decreased in the striatum of forebrain-specific BDNF conditional null mice (Strand et al., 2007), suggesting that it could also be a target of BDNF regulation in the cortex. We chose to more carefully examine Wnt2 as a candidate BDNF-regulated Wnt gene for several additional reasons. First, Wnt2 expression is increased by neural activity in hippocampal neurons (Wayman et al., 2006) and BDNF expression is known to mediate some activity-dependent neuronal processes (Ghosh et al., 1994; Kuczewski et al., 2010). Second, Wnt2 is sufficient to increase dendrite length in developing hippocampal neurons (Wayman et al., 2006) and, similarly, BDNF influences dendrite growth in cortical neurons (Gorski et al., 2003; McAllister et al., 1997; Xu et al., 2000). Third, antidepressant drug treatment can increase Wnt2 expression (Okamoto et al., 2010) and BDNF has been shown to be a crucial mediator of these antidepressants (Adachi et al., 2008; Shirayama et al., 2002). Thus, a variety of functional similarities between BDNF and Wnt2 have been previously described, supporting the possibility of a functional relationship.

**Fig. 4:**
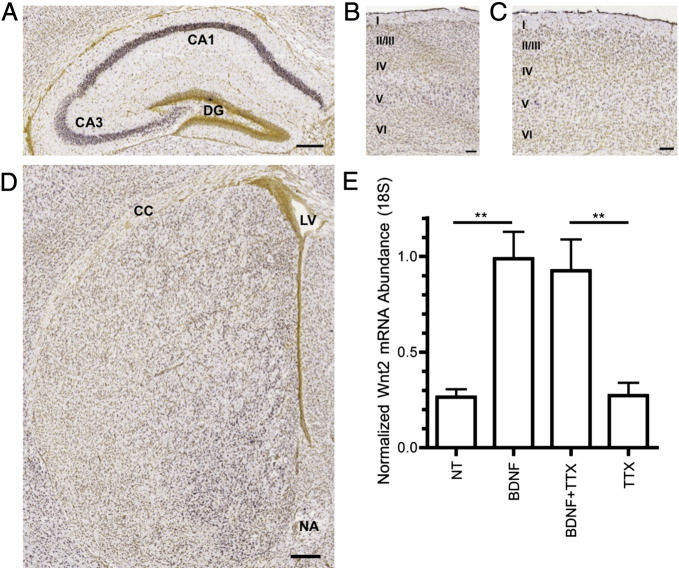
Wnt2 is expressed in the developing mouse brain and its expression in cortical cultures is increased by BDNF. *In situ* hybridization images taken from the Allen Developing Brain Atlas (Allen Developing Mouse Brain Atlas, 2009) showing Wnt2 expression (purple color) in the hippocampus (A), primary motor cortex (B), primary visual cortex (C) and striatum (D) at P14. (E) Quantification of Wnt2 mRNA abundance after treatment with recombinant BDNF, TTX or both, normalized to 18S RNA. **p < 0.01. n = number of wells: NT n = 5, BDNF n = 4, BDNF + TTX n = 5, TTX n = 4. Scale bars: (A, D) 250 μm, (B, C) 100 μm.

We first analyzed the expression pattern of Wnt2 in the brain during periods of increased dendritic spine formation using the Allen Developing Mouse Brain Atlas, a genome-wide mouse brain *in situ* hybridization library (Allen Developing Mouse Brain Atlas, 2009). Wnt2 is expressed in cortex, hippocampus and striatum at P14 (Figs. 4A–C, S1B), when dendritic spine and synapse addition occurs at a high rate *in vivo*. Notably, dendritic spine formation on neurons in each of these brain regions is influenced by BDNF (Baquet et al., 2004; Chakravarthy et al., 2006; English et al., 2012; Kaneko et al., 2012; Rauskolb et al., 2010; Vigers et al., 2012).

To determine whether BDNF regulates Wnt2 expression, we treated DIV10 cultured cortical neurons with recombinant BDNF and used qRT-PCR to determine Wnt2 mRNA abundance (Fig. 4E). Application of recombinant BDNF resulted in an approximate 3-fold increase in the level of Wnt2 mRNA (Fig. 4E). Additionally, co-treatment with tetrodotoxin (TTX), which blocks voltage-gated sodium channels, demonstrated that the BDNF-induced increase of Wnt2 mRNA abundance occurs in the absence of evoked neural activity (Fig. 4E). Together, these data are consistent with the possibility that Wnt2 is a target of BDNF regulation in several brain regions including the cortex, and that BDNF can regulate Wnt2 expression independently of neural activity.

### Wnt2 promotes cortical dendrite growth and dendritic spine formation

**Fig. S4:**
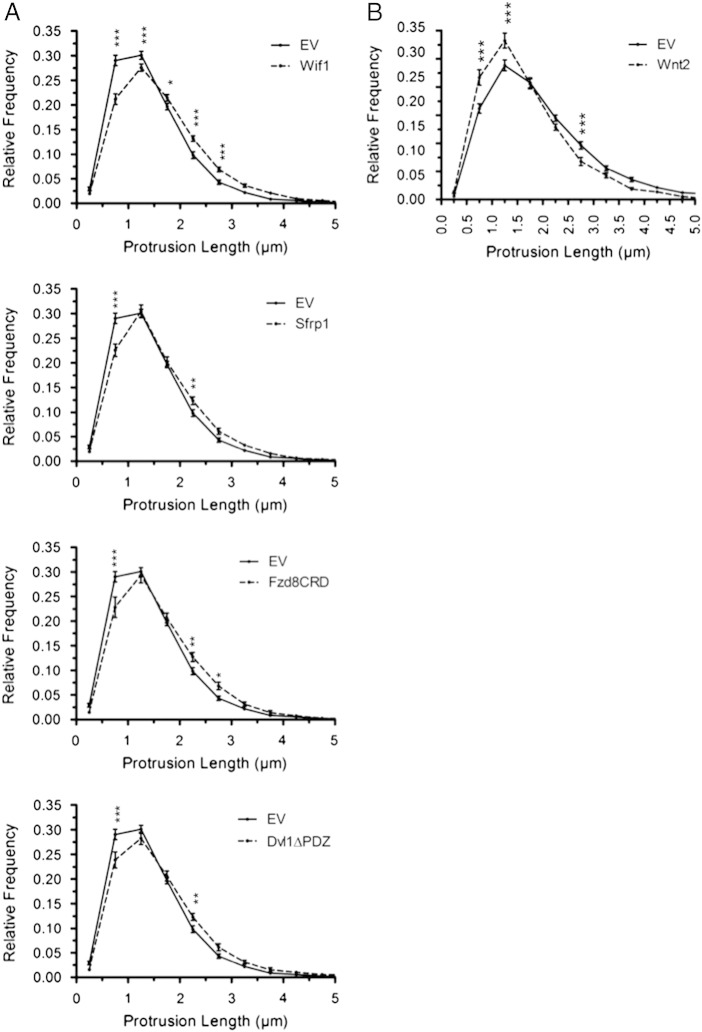
Expression of Wnt inhibitors or Wnt2 shifts the distribution of dendritic protrusion lengths. (A) Relative frequency distributions of dendritic protrusion length comparing Wif1, Sfrp1, Fzd8CRD and Dvl1ΔPDZ expressing neurons to EV control. (B) Relative frequency distribution of protrusion length comparing Wnt2 expressing neurons to EV control. n = number of neurons: EV(A) n = 31, Wif1 n = 34, Sfrp1 n = 23, Fzd8CRD n = 25, Dvl1ΔPDZ n = 25; EV(B) n = 29, Wnt2 n = 25. ***p < 0.001, **p < 0.01, *p < 0.05.

**Fig. 5:**
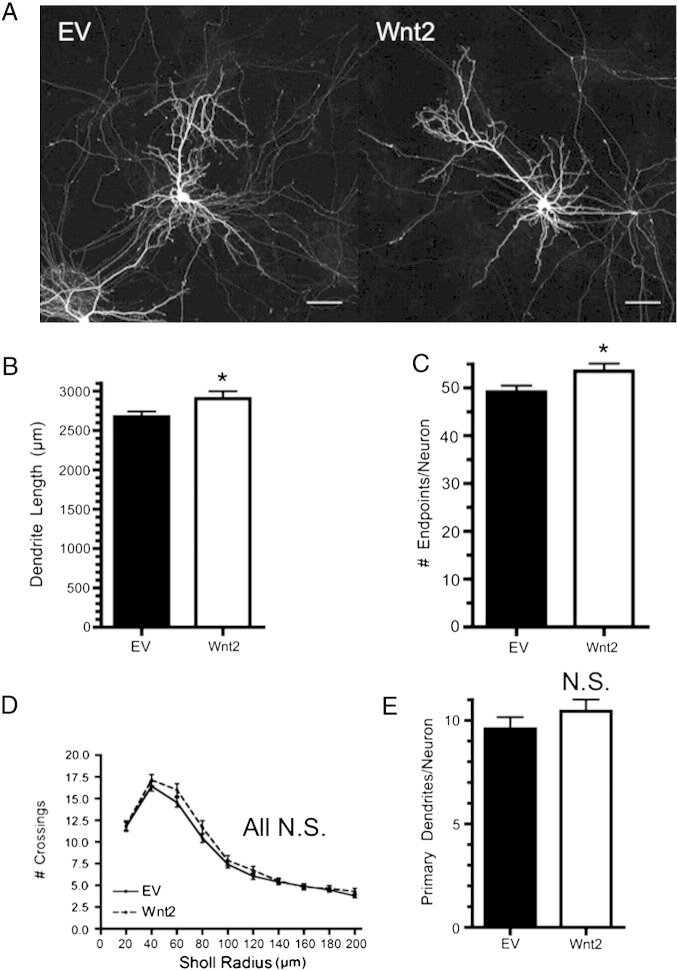
Wnt2 overexpression is sufficient to increase cortical dendrite length. (A) Representative cortical neurons expressing either EV or Wnt2. Quantification of the total dendrite length per neuron (B) and the number of dendritic endpoints per neuron (C) for each treatment. (D) Sholl analysis of dendritic complexity comparing Wnt2 expressing neurons to control. (E) Quantification of the number of primary dendrites per neuron for each treatment. *p < 0.05. n = number of neurons: EV n = 52, Wnt2 n = 44. Scale bar: 50 μm.

**Fig. 6:**
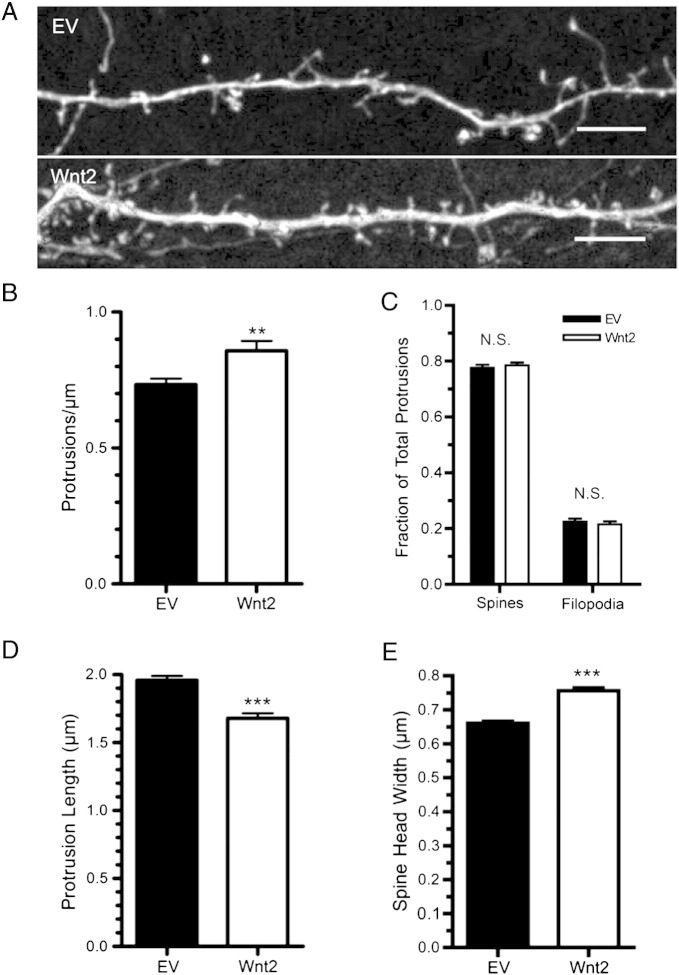
Wnt2 overexpression increases dendritic protrusion density and influences spine shape on cortical neurons. (A) Representative dendritic segments of cortical neurons expressing either EV or Wnt2. (B) Quantification of dendritic protrusion density. (C) Percent of all dendritic protrusions classified as either spines or filopodia. Quantification of average protrusion length (D) and average spine head width (E) for each treatment. ***p < 0.001, **p < 0.01. n = number of neurons: EV n = 29, Wnt2 n = 25. Scale bar: 5 μm.

We next sought to determine whether Wnt2 overexpression by cortical neurons influences their dendritic morphology. Similar to our findings with BDNF, there is less Wnt2 mRNA in our DIV14 primary cortical cultures than *in vivo* at P14 (Fig. S1B). Wnt2 overexpression resulted in a small but significant increase in total dendrite length (Fig. 5A, B), consistent with what has been shown previously for Wnt2 in hippocampal neurons (Wayman et al., 2006). Additionally, Wnt2 expression resulted in a small but significant increase in the number of dendrite endpoints (Fig. 5C). However, Wnt2 did not significantly increase overall dendritic complexity as measured by Sholl analysis, and did not increase the number of primary dendrites (Fig. 5D, E). Images of representative dendrite segments overexpressing Wnt2 are shown in Fig. 6A. Quantification revealed that Wnt2 caused a 17 ± 4% increase in dendritic spine density (Fig. 6B). Additionally, Wnt2 caused a significant decrease in average protrusion length (Fig. 6D) and increased the fraction of short protrusions (Fig. S4E). Further, while Wnt2 expression did not alter the percentage of filopodial spines (Fig. 6F), it did cause a significant increase in dendritic spine head width (Fig. 6C). Together, these data indicate that Wnt2 is sufficient to increase cortical dendritic spine formation and/or stabilization, and may play an important role in promoting dendritic spine maturation.

## Discussion

Our studies indicate that Wnt signaling modulates cortical dendrite growth and dendritic spine formation. In addition, our observations argue that the Wnt signaling system cooperates with the neurotrophin BDNF to regulate dendritic spine formation and maturation. We present evidence indicating that at least one specific Wnt, Wnt2, is regulated by BDNF, and its overexpression in cortical neurons is sufficient to increase dendrite growth and promote dendritic spine formation.

### Wnt inhibition and dendritic spine maturation

We found that a series of different Wnt signaling inhibitors were able to block BDNF-induced increases in dendritic spine density and dendritic spine head width. Additionally, in the absence of BDNF overexpression, these same Wnt inhibitors increased the fraction of immature filopodial protrusions and significantly reduced the size of dendritic spine heads, while only one inhibitor affected dendritic protrusion density. The lack of effects on spine density in the absence of BDNF overexpression is consistent with relatively low levels of BDNF expression in our cultures at the time studied, and with a role for Wnt signaling in promoting maturation of many cortical dendritic spines, only some of which require BDNF for their formation. Our results support a model in which increased BDNF expression stimulates dendritic spine formation, which requires increased Wnt signaling in order for newly formed dendritic spines to mature and stabilize.

Alternatively, it is possible that the secreted Wnt inhibitors used in our studies do not completely inhibit signaling by all of the Wnts present in cortical cultures. Previous studies have indicated that endogenous and non-endogenous secreted Wnt inhibitors vary in the affinity with which they bind and inhibit the activity of Wnt proteins (Carmon and Loose, 2010; Galli et al., 2006). To date, six of the 19 different mammalian Wnt proteins have been shown to be active on neurons (Salinas and Zou, 2008), and three Wnt proteins have specifically been identified as modulators of dendritic spine formation (Ciani et al., 2011; Farias et al., 2009). Because the majority of Wnts and their Frizzled receptors appear to be expressed in the mouse neocortex at P14 (Allen Developing Mouse Brain Atlas), it is possible that multiple Wnts regulate cortical dendritic spine formation. The large repertoire of Wnts and their receptors seems well suited to contribute to complexity in cortical circuit development and plasticity.

### Wnt and neurotrophin signaling interaction during dendritic spine formation

Prior studies have identified intracellular effectors of BDNF that modulate dendrites and synapses. In contrast, our studies support a requirement for secreted Wnt signaling proteins during BDNF-induced dendritic spine formation. This mechanism could rely upon BDNF regulating Wnt expression, or other aspects of Wnt signaling, in order to regulate dendritic spine formation. We report here that BDNF increases the abundance of at least one Wnt mRNA, Wnt2. However, the mechanism by which the abundance of Wnt2 mRNA is regulated remains unclear. Increased Wnt2 mRNA levels could reflect regulation of transcription or mRNA stability. BDNF is known to regulate mRNA translation in neurons (Schratt et al., 2004), and translation can influence mRNA stability. Intriguingly, Wnt2 mRNA is highly translated in striatal neurons (Doyle et al., 2008), perhaps suggesting that the necessity for BDNF in formation of many striatal dendritic spines (Baquet et al., 2004) reflects regulation of Wnt2 translation by BDNF in striatal neurons.

Neural activity is a key regulator of dendritic spine formation (Alvarez and Sabatini, 2007), and it also regulates Wnt gene expression transcriptionally (Wayman et al., 2006), translationally (Gogolla et al., 2009), and post-translationally (Ataman et al., 2008; Chen et al., 2006; Tabatadze et al., 2011). Importantly, activity-mediated regulation of Wnt signaling is required for activity-mediated synapse formation (Chen et al., 2006; Gogolla et al., 2009; Sahores et al., 2010). Further, neural activity increases the secretion of BDNF (Kuczewski et al., 2010) and, in a reciprocal manner, BDNF signaling increases neural activity (Madara and Levine, 2008; Pozzo-Miller, 2006). Interestingly, BDNF signaling is required to induce long-lasting structural dendritic spine plasticity in a paradigm that pairs glutamate uncaging with post-synaptic neural activity (Tanaka et al., 2008), suggesting that neural activity and BDNF coordinately regulate dendritic spine formation. Together with our results, these previous observations suggest an intertwined regulatory relationship between BDNF, neural activity and Wnt signaling in regulation of dendritic spines.

### Directionality of Wnt signaling during BDNF-induced dendritic spine formation

The data we describe here suggest insight into the directionality of Wnt signaling during BDNF-induced cortical dendritic spine formation. We found that the cytoplasmic Wnt inhibitor, Dvl1ΔPDZ, inhibits BDNF-induced increases in dendritic spine formation to a similar degree as the three secreted Wnt inhibitors, Wif1, Sfrp1 and Fzd8CRD (Fig. 3B). This implies that the isolated transfected cells overexpressing BDNF must receive and transduce a Wnt signal in order to add the BDNF-induced dendritic spines. Interestingly, Wnt7a has been suggested to promote hippocampal dendritic spine growth by regulating postsynaptic CamKII signaling (Ciani et al., 2011) while Wnt5a is thought to promote hippocampal dendritic spine formation by regulating postsynaptic clustering of PSD-95 (Farias et al., 2009). Therefore, it is possible that Wnts regulate cortical dendritic spine formation through similar postsynaptic mechanisms. However, this does not preclude the possibility that Wnt signaling also regulates cortical dendritic spines through a presynaptic mechanism.

### Wnt signaling and the relationship between dendritic spines and dendrite arbors

We also found that a series of different Wnt inhibitors can affect elaboration of cortical dendritic arbors, reminiscent of findings with hippocampal neurons (Rosso et al., 2005). Previous work has established that there is a correlation between dendritic spine size and synaptic strength (Matsuzaki et al., 2001; Zito et al., 2009), and studies have shown that blocking Wnt signaling impairs excitatory neurotransmission (Cerpa et al., 2011; Varela-Nallar et al., 2010). We speculate that reduced excitatory synaptic input onto neurons as a result of impaired dendritic spine formation and maturation due to inhibition of Wnt signaling may lead to the reduction in cortical dendritic arbor size and complexity as predicted by the synaptotrophic hypothesis.

### Connections between BDNF and Wnt2 in dendritic spine-associated neuropathologies

Our finding that Wnt2 overexpression in cortical neurons promotes dendritic spine formation, increases dendritic spine head width and decreases dendritic spine length, is similar to the effects of other Wnt proteins on hippocampal dendritic spine formation (Ciani et al., 2011; Farias et al., 2009; Varela-Nallar et al., 2010). Several different anti-depressant drugs increase the expression of Wnt2, and viral-mediated overexpression of Wnt2 in the hippocampus alleviates depressive-like symptoms in some animal models of depression (Okamoto et al., 2010). Loss of hippocampal dendritic spines has been reported in the learned helplessness model of depression (Hajszan et al., 2009), and anti-depressant drug treatment has been shown to increase dendritic spine formation in the hippocampus (Norrholm and Ouimet, 2001) and cortex (Ampuero et al., 2010). Our results suggest that Wnt2 may alleviate depressive-like symptoms by promoting dendritic spine formation. Moreover, multiple studies have shown that BDNF is both necessary and sufficient for anti-depressant drug action (Banasr and Duman, 2008; Castren and Rantamaki, 2010; Duman and Monteggia, 2006; Schmidt et al., 2008; Yu and Chen, 2011), which leads to the interesting possibility that BDNF-mediated regulation of Wnt2 expression may be an important mechanistic link in the alleviation of symptoms during depression.

Wnt2 expression is reduced in the hippocampus and cortex of FMR1 knockout mice, a mouse model for Fragile-X syndrome (Zhang et al., 2009). Fragile-X syndrome is a neurodevelopmental disorder characterized by an overabundance of immature cortical dendritic spines (Antar et al., 2006; Irwin et al., 2001; Nimchinsky et al., 2001). Furthermore, it has been suggested that the specific dendritic spine defect seen in Fragile-X syndrome results from decreased stability of dendritic spine contacts (Cruz-Martin et al., 2010; Pan et al., 2010). Our results showing that Wnt2 increases spine number, increases spine head width and decreases spine length in cortical neurons imply that Wnt2 functions to promote dendritic spine maturation. This finding implicates Wnt2 deficiency as potentially critical in the etiology of the dendritic spine pathology seen in Fragile-X syndrome.

### Evolution and interrelationships between Wnt and neurotrophin signaling

Neural synapses are an evolutionarily ancient structure thought to have evolved in early metazoans (Ryan and Grant, 2009). Similarly, the emergence of the Wnt signaling pathway coincides with the emergence of metazoan life (Croce and McClay, 2008), consistent with the possibility that Wnt signaling plays a fundamental role during synapse formation. In contrast, neurotrophin signaling pathways evolved more recently, with the ancestral neurotrophin believed to have arisen early in vertebrate evolution (Hallbook, 1999). The later evolution of neurotrophins is entirely consistent with the possibility that the newly evolved neurotrophin signaling system was able to recruit the pre-existing Wnt signaling system to regulate interactions at synapses. Indeed, NGF regulates Wnt5 expression during sympathetic neuron axon development (Bodmer et al., 2009). Interestingly, there is also evidence for reciprocal regulation of neurotrophins by Wnts; several Wnts regulate Neurotrophin-3 expression during sensory nervous system development (Patapoutian et al., 1999), and Wnt3a regulates BDNF expression in retinal glial cells (Yi et al., 2012). We suggest that the neurotrophin and Wnt signaling systems mediate a communication dialog between neurons and their innervation targets that shapes the development and plasticity of neural circuitry in the cerebral cortex and elsewhere in the nervous system.

## Experimental methods

### Cortical neuron cultures

Cortices were dissected from postnatal day 0–1 CD1 mouse pups and incubated in papain (Worthington, Lakewood, NJ) for 45 min at room temperature. The tissue was triturated to obtain a single-cell suspension, and 2.5 × 10^6^ cells/cm^2^ were plated into 12-well cell-culture dishes coated with poly-d-lysine (Sigma-Aldrich, St. Louis, MO). Cells were grown initially in DMEM (Life Technologies Corporation, Carlsbad, CA) supplemented with 10% Fetal Bovine Serum (Atlanta Biologicals, Lawrenceville, GA) and Penicillin–Streptomycin (Life Technologies). After 24 h, the culture medium was changed to Neurobasal-A medium (Life Technologies) supplemented with B27 (Life Technologies), Glutamax (Life Technologies), Penicillin–Streptomycin (Life Technologies), and 5-Fluorodeoxyuridine (Sigma-Aldrich) to prevent glial cell proliferation. Cultures were maintained at 5% CO_2_ for 10 days *in vitro* (DIV) before beginning experimental procedures, with half of the medium changed on DIV9. For all experiments, neuron cultures were fixed with HEPES-buffered (Thermo Scientific, Waltham, MA) 4% paraformaldehyde (Sigma-Aldrich), 4% Sucrose (Thermo Scientific) solution in PBS (pH 7.4). After fixation, neuron cultures were mounted in Fluoromount-G (Southern Biotech, Birmingham, AL) for subsequent analysis.

### Neuron transfection

Neuron cultures were transfected on DIV10 using Lipofectamine 2000 (Life Technologies) according to manufacturer's instruction. Briefly, plasmid DNA was prepared for transfection at a DNA (μg):Lipofectamine (μL) ratio of 1:3 in serum-free Neurobasal-A (Life Technologies). In all experiments, 1.5 μg of total plasmid DNA was transfected per well of a 12-well cell-culture dish, and all plasmids used the CMV promoter to drive gene expression. For each transfection, 500 ng of plasmid encoding each protein of interest was used, with the exception of experiments utilizing the tet-inducible expression system, in which case 400 ng of pCMV-TRE-tight-GFP and 100 ng of pCMV-rtTA were used. This strategy of using consistent plasmid amounts in transfection mixes has been previously used to achieve consistent levels of expression of a plasmid-encoded gene across transfections (Margolis et al., 2010). An empty vector plasmid containing the CMV promoter was used in each transfection to bring the total amount of plasmid DNA in each transfection to 1.5 μg. Cultures were transfected for 2 h and then the transfection medium was replaced with fresh culture medium. Transfection efficiency was approximately 5–10% using this method.

### Expression plasmids

pCMV-BDNF was constructed by inserting the open reading frame for murine BDNF into the pEGFP-N1 backbone (Clontech, Mountain View, CA). pBR22-Wif1 expresses murine Wif1 and was a generous gift from Dr. Ken Iwatsuki (Ajinomoto Co., Tokyo, Japan). pCS2 +-Sfrp1 (Addgene plasmid 16693, Cambridge, MA) expresses murine Sfrp1 (Randall Moon Lab). pRK5-mFzd8CRD-IgG (Addgene plasmid 16689) expresses a fusion protein consisting of the extracellular domain of the murine Frizzled-8 protein fused with the human immunoglobulin heavy chain (Semenov et al., 2001). pCS2 +-mDvl1ΔPDZ-HA expresses a C-terminally HA tagged murine Dishevelled-1 with a deletion of amino acids 276–336 (Rosso et al., 2005). pTRE-tight-BDNF and pTRE-tight-Wnt2 were constructed by inserting the ORFs for murine BDNF and murine Wnt2, respectively, into the pTRE-tight backbone (Clontech). pTRE-tight-EGFP was constructed by inserting the EGFP ORF from pEGFP-N1 (Clontech) into the pTRE-tight backbone. pCMV-rtTA was constructed by inserting the ORF for the reverse-tet-Transactivator protein from pTRIPZ (Thermo Scientific) into the pEGFP-N1 backbone from which the EGFP coding region was deleted. BDNF, GFP and Wnt2 expression was induced from pTRE-tight plasmids by adding 1 μg/mL doxycycline (BD Biosciences, San Diego, CA) to the culture medium. pEGFP-N1 was used to express cytoplasmic GFP.

### Image acquisition and analysis

Dendritic protrusion, primary dendrite and nuclear c-Fos images were collected using Metamorph software (Molecular Devices, Downington, PA) and a Nikon Eclipse TE2000-U microscope (Nikon, Melville, NY) fitted with a spinning disk confocal system (Solamere Technology Group, Salt Lake City, UT) and a Cascade II 16-bit EMCCD camera (Photometrics, Tucson, AZ). Dendritic arbor images were collected using Zen software (Carl Zeiss, Germany) and a Zeiss LSM 510 Meta confocal system (Carl Zeiss, Germany).

To quantify dendritic protrusion density and length, 8–10 neurons per coverslip from a total of 3–4 coverslips per treatment were imaged using a 1.40NA 100 × objective (Nikon). Neurons that had a pyramidal-shaped cell body and a clear apical dendrite were chosen for analysis. 5 dendritic segments (3 apical segments and 2 basal segments) per neuron were imaged in 0.2 μm Z-steps. Z-stacks were then loaded into ImageJ (National Institutes of Health, http://rsb.info.nih.gov/ij) for analysis. Total dendritic protrusion density was measured using uncompressed Z-stacks. All dendritic protrusions less than 5 μm in length were counted and then quantified as the number of dendritic protrusions per μm of dendrite length. Average apical and basal protrusion densities for each neuron were calculated from the apical and basal segments, respectively. The total protrusion density per neuron was calculated as the average of the apical and basal protrusion density.

In order to quantify the percentage of spine and filopodial protrusions, and to measure dendritic protrusion length and dendritic spine head width, Z-stacks were flattened using the stack focuser plugin for ImageJ (n × n Kernel = 11). To group dendritic protrusions into categories, they were classified as either “spines” or “filopodia.” “Spines” were defined as dendritic protrusions that possessed a discernable spine head distinct from the dendritic spine neck. The exception was spines that were less than 0.5 μm in length that were at least 0.5 μm wide. Using these criteria, the “spine” category of all protrusions encompassed three spine morphologies (thin, mushroom and stubby). “Filopodia” were defined as dendritic protrusions that lacked a discernable spine head as distinct from the dendritic spine neck. Protrusions less than 0.5 μm in length and 0.5 μm in width were classified as “filopodia” in order to distinguish short filopodia from stubby spines.

Protrusion length was measured from protrusions emanating in a perpendicular direction from the dendrite shaft in the X/Y plane using the freehand line drawing tool in ImageJ. Protrusion length was defined as the distance between the dendrite shaft and the tip of the protrusion. Protrusions emerging either above or below the dendrite were not measured in order to minimize the effects of the Z-projection on length. The length of all protrusions was measured in this manner, including both dendritic spines and filopodia. Spine head width was measured for protrusions classified as “spines,” and was defined as the widest portion of the dendritic spine head.

To measure the number of primary dendrites, the cell body of the neurons used each to measure dendritic protrusions was imaged in 0.5 μm Z-steps. Uncompressed Z-stacks were analyzed in ImageJ. Primary dendrites were defined as all neurites that emerged directly from the cell body of the neuron.

To quantify dendritic arbors, 10–20 neurons per coverslip from a total of 3–4 coverslips per treatment were imaged using a 0.8NA 20 × objective (Carl Zeiss) in 1.0 μm Z-steps. Using ImageJ, Z-stacks were flattened using a Max-point Z-projection. Dendritic arbors were traced using the NeuronJ plugin for ImageJ (Eric Meijering, Biomedical Imaging Group, Erasmus MC, University Medical Center, Rotterdam, Netherlands). NeuronJ settings were as follows: Neurite appearance: Dark; Hessian smoothing scale: 2.0; Cost-weight factor: 0.7; Snap window size: 5 × 5; Path search window: 2500 × 2500; Tracing smoothing range: 5; Tracing subsampling factor: 5; Line width: 1. Dendritic arbor traces were skeletonized to a pixel width of 1.0 and then converted into a binary image. Total dendrite length was calculated by measuring the total pixel count for each tracing and then converting the pixel count into a length measurement (1.0 μm dendrite length per 2.27 pixels) for each neuron that was traced. Sholl analysis was performed on the skeletonized dendritic arbor traces using the automated Sholl analysis plugin for ImageJ (Ghosh Lab, University of California, San Diego). Sholl analysis settings were as follows: Starting Radius: 20 μm; Ending Radius: 200 μm; Radius Step Size: 20 μm; Radius Span: 0.00; Span Type: median. Dendrite endpoints were also measured in ImageJ and were defined as any time a dendrite branch terminates. All imaging and analysis was performed in a blinded fashion with respect to neuron treatment.

### Quantitative reverse transcriptase PCR

To test for the regulation of Wnt2 mRNA expression by BDNF, DIV10 cultured neurons were treated for 4 h with either 50 ng/mL recombinant BDNF (Millipore, Billerica, MA), 4 μm tetrodotoxin (TTX, Sigma Aldrich), or both BDNF and TTX in combination. For experiments investigating the temporal expression profile of BDNF and Wnt2 mRNA, cultured neurons were harvested on DIV0, DIV10 and DIV14. Additionally, neocortex was dissected from P14 mouse brain. Total RNA for all qRT-PCR experiments was prepared using Trizol reagent (Life Technologies) according to the manufacturer's instructions. RNA was converted to cDNA using the iScript cDNA synthesis kit (BIO-RAD, Hercules, CA) according to the manufacturer's instructions. Quantitative real-time PCR analysis was performed using an Applied Biosystems 7500 Fast Real-Time PCR System (Life Technologies). BDNF and Wnt2 mRNA abundances were normalized to 18S RNA. Reactions were performed in triplicate, in two separate experiments.

### Measurement of c-Fos induction by BDNF

To measure the induction of c-Fos expression by BDNF, cortical neurons were co-transfected on DIV10 with pTRE-tight-BDNF, pTRE-tight-EGFP and pCMV-rtTA. To test the effects of Wnt inhibition on BDNF-induced c-Fos expression, neurons were additionally co-transfected with plasmids expressing one of the four Wnt inhibitors or an empty vector control. Neurons were allowed to express the inhibitors for 2DIV, and then 1 μg/mL doxycycline was added to the culture medium to induce BDNF expression. 12 h after induction of BDNF, neurons were fixed and immunostained for c-Fos. c-Fos immunostaining was performed using Rabbit polyclonal SC-52 anti-c-Fos primary antibody (Santa Cruz Biotechnology, Santa Cruz, CA), and Alexa Fluor Goat-anti-Rabbit 555 secondary antibody (Life Technologies). Neuronal nuclei were stained using DAPI.

To quantify c-Fos induction, 10 neural cell bodies per treatment were imaged in 0.5 μm Z-steps using a 1.40NA 100 × objective (Nikon). Laser settings were kept consistent throughout image acquisition to allow for subsequent quantitation of nuclear c-Fos intensity. In order to eliminate sample bias and to ensure that neurons were not selected for imaging based on observed c-Fos immunoreactivity during image acquisition, neurons were selected for imaging using only their morphology as indicated by GFP fluorescence. The first 10 neuronal cell bodies meeting our morphological criteria (pyramidal-shaped body, one distinct apical dendrite) were imaged in this manner. c-Fos expression was quantified by loading uncompressed Z-stacks into ImageJ. The DAPI fluorescence was used to outline a region of interest around the nucleus in a single image plane in which the nucleus was at maximum size. The integrated density within this region was measured in the corresponding image plane displaying c-Fos immunoreactivity. Normalized nuclear c-Fos intensity was calculated by dividing the integrated density by the area of the ROI.

### Statistical analysis

Statistical significance for experiments comparing two populations was determined using a two-tailed unpaired Student's *t*-test. For experiments comparing three or more populations, a One-way ANOVA with Tukey's post-hoc test was used. For spine length frequency distributions and Sholl analysis distributions, statistical significance was determined using a Two-way ANOVA with a Bonferroni post-test to compare means at individual data points. All statistical analyses were performed using Graphpad Prism (Graphpad Software, Inc., La Jolla, CA). All data are presented as the mean ± SEM with n = number of neurons. Data presented here are representative of results from at least two separate experiments.

The following are the supplementary data related to this article.
